# Burns

**Published:** 1864-06

**Authors:** 


BURNS.—The best treatment of burns and scalds was introduced by Dr. Kentish, of Bristol, about half a century ago, and consists in the use of stimulating applications to the injured surface. Dr. Skey has fully tested this treatment for a number of years, in a great number of cases, and feels convinced after using all other methods, which are supposed to soothe, allay, or calm the pain, and believes that any one trying the two plans, will adopt the stimulating treatment. As an instance he gives the following: Five were brought into the hospital at one time, severely burnt by an explosion of gas. One died immediately, the remaining four were badly burnt about the face, chest, and arms. The face and chest of each man were washed with a solution of ten grains of nitrate of silver. To the arms was applied the celebrated Carron, or boiled oil. Twenty-four hours elapsed, and on inquiry whether the patients were suffering any pain, each made the same reply, "I am easy everywhere, except in the hands and arms." The oil was removed, the solution was applied, and relief followed immediately. The solution may be applied at any time, so long as the pain remains. Ten or fifteen grains to an ounce of water for an adult,—five to seven for a child, is the strength employed.—*London Lancet.*

